# Food-induced anaphylaxis following skin prick testing: a case report and literature review

**DOI:** 10.3389/falgy.2026.1796432

**Published:** 2026-05-29

**Authors:** E. Novembre, F. Mori, C. Mastrorilli, Stefania Arasi, Simona Barni, Lucia Caminiti, Riccardo Castagnoli, Mariannita Gelsomino, Mattia Giovannini, Angela Klain, Lucia Liotti, Luca Pecoraro, Francesca Saretta, Gian Luigi Marseglia

**Affiliations:** 1 Department of Health Sciences, University of Florence, Florence, Italy; 2 Allergy Unit, Meyer Children's Hospital IRCCS, Florence, Italy; 3 Pediatric Department, Pediatric Hospital Giovanni XXIII, AOU Policlinic of Bari, Bari, Italy; 4 Translational Research in Paediatric Specialities Area, Division of Allergy, Bambino Gesù Children's Hospital, IRCCS, Rome, Italy; 5 Department of Human Pathology in Adult and Development Age “Gaetano Barresi,” Allergy Unit, Department of Pediatrics, AOU Policlinico Gaetano Martino, Messina, Italy; 6 Department of Clinical, Surgical, Diagnostic and Pediatric Sciences, University of Pavia, Pavia, Italy; 7 Pediatric Clinic, Fondazione IRCCS Policlinico San Matteo, Pavia, Italy; 8 Department of Life Sciences and Public Health, Pediatric Allergy Unit, University Foundation Policlinico Gemelli IRCCS, Catholic University of the Sacred Heart, Rome, Italy; 9 Department of Woman, Child and General and Specialized Surgery, University of Campania Luigi Vanvitelli, Naples, Italy; 10 Pediatric Unit, Department of Mother and Child Health, Salesi Children's Hospital, Ancona, Italy; 11 Department of Experimental Medicine Pediatric Section, University of Salento Hospital “Vito Fazzi,” Lecce, Italy; 12 Pediatric Department, Latisana-Palmanova Hospital, Azienda Sanitaria Universitaria Friuli Centrale, Udine, Italy

**Keywords:** anaphylaxis, atopic dermatitis (AD), generalized allergic reactions (GARs), prick-to-prick (PtP), skin prick test (SPT), specific serum IgE (sIgE), systemic reactions (SRs)

## Abstract

A case of legume-induced anaphylaxis following prick-to-prick (PtP) testing in a 2.5-year-old girl is described. Very few cases of food-induced anaphylaxis following skin prick testing are reported in the literature. Fish and nuts are the most commonly implicated allergens; however, milk, egg, legumes, and vegetables may also be involved. Some potential risk factors have been suggested, including young age, concomitant atopic dermatitis (AD) or asthma, large diameter of the skin test reaction, a history of previous anaphylaxis, and the use of fresh food allergens. The most convincing risk factor reported is the use of PtP testing with fresh, multiple, and often cross-reactive food allergens. When indicated, diagnostic testing should be performed in separate sessions, and the number of PtP tests with fresh foods should therefore be minimized. Physicians should be aware of the potential risk of severe reactions with PtP in certain diagnostic circumstances; therefore, emergency equipment and medications for the treatment of allergic reactions should always be available.

## Highlights

1. Food skin prick testing is generally safe; however, very rare systemic reactions, including anaphylaxis, may occur, particularly with food allergens and in young children.

2. Prick-to-prick testing with fresh foods carries a higher risk of systemic reactions compared with testing with commercial extracts.

3. Fish and nuts are the most commonly implicated allergens, although milk, egg, legumes, and vegetables may also be involved.

4. Key risk factors for adverse reactions include young age, polysensitization, large wheal size, and performing multiple simultaneous prick-to-prick tests with cross-reactive allergens. Performing PtP in multiple sessions for at-risk individuals is highly recommended.

5. Emergency equipment and medications must always be immediately available when performing food skin testing.

## Background

Skin prick testing (SPT) is commonly used to demonstrate immediate IgE-mediated allergic reactions (1). The prick-to-prick (PtP) test is a specialized variation of the conventional SPT. Unlike standard SPTs, which utilize commercially available allergen extracts, the PtP test involves pricking a food sample and immediately using the same lancet to prick the patient's skin. This method is particularly useful for testing allergens that may degrade in commercial extracts or when fresh food allergens contain labile proteins that are difficult to standardize in commercially prepared solutions (2). PtP testing can also serve as a cost-effective alternative in settings where commercial extracts are unavailable, providing a practical solution for assessing regional or less common food allergens (3). Both SPT and PtP testing with fresh foods are commonly used to diagnose IgE-mediated food allergies and are considered safe procedures (1). PtP-induced systemic reactions, including anaphylaxis, are rare but have been reported, particularly in young children and in patients with polysensitization or large skin test reactions (4). PtP testing with multiple fresh foods may increase the risk of systemic reactions due to a higher allergen load and additive histamine release from cutaneous mast cells (5).

We report a case of anaphylaxis occurring in a young child after PtP testing with multiple fresh legumes.

## Case presentation

A 2.5-year-old girl presented to our outpatient hospital office with a history of vomiting at 8 months of age after eating, for the first time, one-fourth teaspoon of cooked brown lentils. At 10 months, she developed redness around the mouth and cough after eating a similar amount of lentils. Subsequently, at 12 months, she experienced contact erythema on her right arm without systemic symptoms after contact with cooked peas and chickpeas; as a result, her parents eliminated all legumes from her diet. Both parents had a history of rhinoconjunctivitis and fruit allergy. During the visit no other significant clinical findings were noted in her personal history or on physical examination. PtP testing was performed in accordance with practice guidelines (1), using single-use prick lancets (3). Histamine (10 mg/mL) and normal saline were used as positive and negative controls, respectively. A positive result was defined as a wheal measuring ≥3 mm greater than the negative control read after 15 min.

SPT with commercial extracts (Lofarma, Milano, Italy) of cow's milk, egg, cod, peanut, soy, and wheat, along with positive and negative controls, was performed on the right forearm to screen for food allergies, given the parental history of rhinoconjunctivitis and food allergy; positive results were observed for egg (4 mm) and peanut (3 mm), with histamine measuring 4 mm and the control 0 mm. However, upon subsequent re-evaluation of the clinical history, no reactions were reported after ingestion of either egg or peanut. As commercial extracts for legumes were not available in the clinical setting, PtP testing with legumes was performed after 5 days, and positive results were observed for all tested allergens (bean 9 mm, lentil 10 mm, lupin 7 mm, and chickpea 9 mm). Approximately 10 min after PtP testing, the child developed an urticarial rash on her face, neck, and arms, along with cough and mild expiratory wheezing without the use of accessory muscles. The respiratory rate was 38 breaths per minute, heart rate was 130 beats per minute, and SaO_2_ was 98%. Antihistamines, oral corticosteroids, and salbutamol via an aerochamber were administered.

Wheezing resolved within a few minutes, and all other symptoms resolved within 1 h. *In vitro* allergen component testing [ALEX test; Macroarray Diagnostics (MAD), Vienna] was subsequently performed, yielding the following positive results (kUA/L): Peanut (Ara h 1: 6.93, Ara h 3: 3.42, Ara h 9: 0.70); chickpea (Cic a: 4.50); lentil (Len c: 9.03); pea (Pis s: 5.63); lupine (Lup a: 2.49); maize (Zea m14, nsLTP: 1.20); apple (Mal d 3, nsLTP: 4.0); peach (Prup 3, nsLTP: 2.21); celery (Api g 1, PR10: 1.92); carrot (Dau c 1, PR10: 2.21); egg (Gal d yolk: 0.56); oyster (Ost e: 1.25); salmon (Sal s 1, parvalbumin: 1.23); mackerel (Sco s: 1.22; Sco s 1, parvalbumin: 0.72); mugwort (Art v3, nsLTP: 4.38); dog (Can f1, lipocalin: 5.16); cat (Fel d 7, lipocalin: 1.08). Total IgE was 200 kU/L.

## Materials and methods

Articles identified through a PubMed search using the terms “skin prick test,” “prick-to-prick,” [AND] “prick by prick” [AND] “systemic reactions” [OR] “anaphylaxis” [OR] “generalized adverse reactions” were included in this narrative review. All articles published from 1980 to December 2025 were considered. Eligible study designs included clinical trials, narrative and systematic reviews, retrospective and prospective observational studies, brief communications, and case reports. Studies were excluded if they contained outdated data, had poor methodological quality, or reported irrelevant outcomes. The initial screening of titles identified potentially relevant studies, followed by screening of abstracts and then full paper reviews.

## Discussion

The present case report describes a young girl who presented with a history of vomiting, episodes of redness around the mouth, and cough following ingestion of lentils, as well as contact erythema without systemic symptoms after expose to peas and chickpeas. During the initial diagnostic evaluation, SPT with common food allergens (cow's milk, egg, cod, peanut, soy, and wheat) was positive for egg and peanut; however, both were normally tolerated in the diet.

A few days after SPT, PtP testing with legumes was performed, showing large positive reactions to all tested allergens; approximately 10 min after testing, the patient developed an anaphylactic reaction. Anaphylaxis was diagnosed according to GA^2^LEN 2024 Anaphylaxis Definition Consensus (6).

*In vitro* testing revealed sIgE to legumes, egg, and other allergens (pollens, dog, and cat). In subsequent anamnesis only sensitization to cats was associated with a contact erythema following exposure to the grandmother's cat.

Legumes, particularly peanut and soybean, are common causes of allergy, especially in childhood (7). Among other legumes, lupine, pea, and lentil are responsible for allergic reactions and, in some cases, anaphylaxis (8).

Legume allergy is frequently reported in children from the Mediterranean region. In a cohort of 54 children with allergic reactions following legume exposure, the onset of allergic reactions occurred at approximately 2 years of age. SPT results were positive for at least three legumes in 38 of 54 children (70%), indicating a high degree of cross-sensitization. Among these, lentil allergy was the most frequently diagnosed (9).

In a study of 22 Spanish children with lentil allergy, based on food challenges or a convincing history of anaphylaxis, along with positive skin tests and/or specific serum IgE (10), the most frequent symptoms were oropharyngeal symptoms and acute urticaria. Four patients experienced anaphylaxis. In 18 patients, symptoms following lentil ingestion started before the age of 4 years (median age, 2.7 years).

Therefore, anaphylaxis can occur in patients with lentil allergy, even at a very young age. Symptoms may also occur via inhalation. Notably, one case of anaphylaxis due to inhalation of steam from cooked lentils has been reported (11).

## Food-induced anaphylaxis following SPT

SPT is widely used in the diagnosis of allergic diseases, as it is considered a quick and safe tool in both adults and children (1). However, some systemic reactions (SRs) can occur (12, 13).

Valyasevi et al. (14), in a retrospective study of 16,505 patients undergoing skin puncture testing for aeroallergens, antibiotics, and latex, reported that 5 patients (0.03%) experienced systemic reactions after SPT alone.

Liccardi et al. (15) reviewed the literature from 1980 to 2005 and found 17 non-fatal cases of anaphylaxix related to skin testing: of these, five were caused by inhalant allergens, two were caused by foods, and the others were caused by drugs, latex, and tetanus toxoid. The authors suggested to pay special attention to very young children, patients with a history of previous systemic/anaphylactic reactions, and those with a high degree of skin reactivity.

Devenney et al. (16), in a retrospective 3-year study (1996–1998) of medical records of 1,152 children, reported six cases of generalized reactions, all in infants younger than 6 months of age with AD and positive SPT results to fresh foods.

Karkin et al. (17) retrospectively studied 1,852 children, with a median age of 31 months, who underwent SPT and/or PtP testing with fresh food allergens. No local reactions or SRs were observed after SPT. Among those tested with PtP, three patients (0.16%) experienced SRs—including one case of anaphylaxis after PtP testing with legumes (0.05%), while the remaining cases presented with angioedema.

In a 3-year prospective study, Norman and Falth-Magnusson (18) reported 14 generalized reactions among 5,908 Swedish children (median age 6.0 years) undergoing SPT. In seven cases (0.12%), symptoms were classified as generalized allergic reactions (GARs) requiring antiallergic medication. Seven were classified as vasovagal reactions, as these patients showed compatible signs and symptoms. Five of the seven children presented with GAR after SPT with food allergens, and two after SPT testing with inhalant allergens. Among the children who presented with GAR after food SPT, only two of five exhibited anaphylactic symptoms. In this study, the risk of developing generalized allergic reactions was higher in young patients and in those with active eczema.

A prospective study conducted in the United States (19) evaluated SRs to skin testing in 1,456 patients (age range 13–70 years, average age 40.6 years). Among those who underwent skin testing, SRs occurred in 3.6% of patients (0.4% following SPT and 3.2% following intradermal testing), all of whom showed prompt improvement after intramuscular epinephrine administration in the deltoid.

In another 6-year prospective study from the United Kingdom (20), the incidence and features of systemic reactions to SPT were evaluated. A total of 31,000 patients underwent SPT with inhalant commercial extracts, venom extracts, fresh foods, or drugs, according to clinical history. Twenty-four patients (mean age 23.5 years) experienced SRs. The SRs to SPT rate was 0.077%. Foods were the most common allergens causing SRs (accounting for 18 cases, mainly involving nuts), followed by aeroallergens (three cases), and single cases related to wasp venom and piperacillin/tazobactam. All 18 cases of food-induced SRs following SPT were treated only with antihistamines, while three patients also required epinephrine and/or steroids. An association was observed between a history of SRs and larger skin test reactions. The prevalence of SRs or GARs following SPT in prospective studies is low, ranging from 0.07% to 0.4% (Table 1).

**Table 1:** Prevalence of systemic reactions following SPT in prospective studies.

Author, year	Adults/children	Number of patients	Systemic reactions, *n* (%)	Severe reactions
Bagg, 2009 (18)	A	1,456	6 (0.4)	All were treated with epinephrine
Sellaturay, 2015 (19)	A + C	31,000	42 (0.077)	Not reported
Norman and Falth-Magnusson, 2009 (17)	C	5,908	7 (0.12)	Two were treated with epinephrine

Several factors have been suggested to identify at-risk subjects, such as young age and concomitant eczema (15, 17), large skin test reactions and asthma (19), and a history of previous anaphylaxis (4); however, the most convincing risk factor reported is the use of PtP testing with multiple fresh food allergens. In fact, the incidence of SRs is higher among those undergoing PtP testing compared with those undergoing SPT alone (0.16 vs. 0%) (16). PtP testing with fresh foods was found to have a higher risk of GARs than testing with commercial food extracts (18, 21). Anyway, the use of fresh foods can increase the sensitivity of testing, as they contain allergens that may be degraded or excluded during the preparation of allergen extracts (e.g., thermolabile or lipophilic allergens) (22). The risk of GARs with PtP testing may be higher in polysensitized individuals (4). Performing multiple tests simultaneously may increase the summation effect of cross-reactive allergens, leading to increased histamine release (5). Reducing the number of PtP tests is therefore recommended when using fresh, cross-reactive foods in the diagnosis of food allergy (4).

In the present study, only a few case reports of SPT-induced anaphylaxis were identified (Table 2). Fifteen cases were associated with PtP testing with fresh foods, while two cases were attributed to commercial extracts (23, 24). Fish was the more frequently implicated allergen (five cases) (23–27), followed by nuts (four cases) (28–31), legumes (two cases, including the present case) (16), egg (one case) (17), milk (one case) (17), and kiwi (one case) (24). A mix of allergens was found in three cases: egg/milk/fish; egg/milk (15); and avocado, cantaloupe, and watermelon (32).

**Table 2:** Case reports of food-induced anaphylaxis following SPT.

Author, year	Food-induced anaphylaxis following SPT	Age (years)/sex	Symptoms of anaphylaxis	Treatment of anaphylaxis	Previous anaphylaxis	Comorbitities
a) Children younger than 18 years						
Devenney, 2000 (15)	1 case: egg, milk, fish (PtP)	5 months/F	Urticaria and wheezing	Epinephrine SC	No	Eczema
	1 case: egg, milk (PtP)	5 months/M	Urticaria, hoarseness, and cough	Epinephrine SC	No	Eczema
Norman and Falth-Magnusson 2009 (17)	1 case: milk (PtP)	6 months/M	Generalized erythema, severe pruritus, itchiness, red conjunctiva, and vomiting	Betamethasone per os	No	Eczema
	1 case: egg (PtP)	5/M	Sore throat, hoarseness, became pale, started coughing, and went into a cold sweat	Betamethasone per os	No	Eczema
Pitsios, 2010 (23)	1 case: fish (SPT with commercial extracts and many species)	5/F	Generalized pruritus, nausea, intense stomach pain, loss of consciousness, hypotension, and undetectable heart rate	Epinephrine IV and intravenous fluids; methylprednisolone and cetirizine (drops) per os were also given as soon as she regained consciousness.	No	Asthma, atopic dermatitis, and perennial rhinoconjunctivitis
Tosca, 2012 (30)	1 case: pine nut (PtP)	2/M	Nasal and conjunctival itching, asthenia, nasal congestion, cough, and dyspnea	epinephrine aerosol, parental chlorpheniramine and steroidal (betamethasone)	Yes	No
Haktanir, 2016 (26)	1 case: fish (PtP with many species)	4/F	Urticarial rash on her face, neck, and upper trunk; angioedema over her right auricle; cough; wheezing; and anxiety	Epinephrine IM, nebulized salbutamol, and oxygen	No	No
Karkin, 2024 (16)	3 cases of generalized reactions, including 1 case of anaphylaxis. 2 cases to legumes (PtP)	9/not reported	Not reported	Epinephrine IM, nasal oxygen, antihistamine IV, steroid IM, inhaled bronchodilator	Yes	No
Present case	Legumes (PtP)	2.5/F	Urticaria, dry cough, and wheezing	Antihistamine, corticosteroids per os, and salbutamol spray	Yes	No
b) Subjects >18 years						
Novembre, 1995 (24)	1 case: kiwi (PtP)	57/M	Erythema of the face, cough, dyspnea, hypotension, and itchy throat	Antihistamines and steroids per os	Yes	Rhinoconjunctivitis
	1 case: fish (SPT with many species)	29/M	Severe dyspnea and collapse	Epinephrine SC, steroids IV, and antihistamines IM	Yes	No
Van de Scheur, 2004 (28)	1 case: pine nut (PtP)	20/F	Skin reaction, acute dyspnea, angioedema, and circulatory collapse	Clemastine IM, epinephrine IM, and oxygen via a mask	Yes	Not reported
Senna, 2005 (29)	1 case: brazil nut, walnut (PtP)	18/M	Dyspnea, facial angioedema, and hypotension (max = 90 mmHg)	Clorpheniramine IM, epinephrine IM, corticosteroid IV, oxygen, and inhaled albuterol plus ipratropium	Yes	No
Pitsios, 2009 (25)	2 cases of generalized reactions, including 1 case of anaphylaxis: fish (PtP)	28/M	Giant urticaria and tachycardia	Cetirizine drops and methylprednisolone per os	Yes	Eczema, rhinitis, and asthma
Hernández-Moreno, 2017 (32)	1 case: avocado, cantaloupe, carrot, and watermelon (PtP)	47/F	Generalized pruritus, erythema, and dyspnea	Epinephrine IM, salbutamol, hydrocortisone IV, and oxygen	Yes	Rhinoconjunctivitis
Alnaes, 2020 (31)	1 case: peanut (PtP)	29/M	Urticaria, dry cough, shortness of breath, and wheezing	Epinephrine IM, salbutamol via an inhalation chamber, and desloratadine and prednisolone per os	Yes	Eczema
Cherrez-Ojeda, 2022 (27)	1 case: fish (PtP)	23/F	Generalized rash, sore throat and cough, difficulty breathing, wheezing, and tachypnea	Epinephrine IM, clemastine IV, salbutamol nebulization, and oxygen	No	Rhinitis and asthma

PtP, prick-to-prick; SC, subcutaneous; IM, intramuscular; IV, intravenous.

Nine patients with SPT-induced anaphylaxis were younger than 18 years; a history of anaphylaxis was recorded in 10 cases, and comorbidities were present in 10 cases. Epinephrine was administered in 12 cases (six adults and six children), delivered IM in seven cases, SC in three cases, IV in one case, and via nebulization in one case (Table 2).

In the present case of anaphylaxis, epinephrine was not administered, despite being indicated (6). This highlights the need for more efforts to disseminate and to promote the appropriate and timely use of epinephrine in all clinical settings.

This case represents the second reported case of legume-induced anaphylaxis following PtP testing and exhibited the same symptoms (urticaria, cough, and wheezing) as the only similar case previously described (16). Both cases occurred in children and had similar risk factors (multiple PtP tests with fresh foods, large skin test reactions, positivity to multiple cross-reactive allergens, and a history of previous anaphylaxis).

In conclusion, skin prick testing is widely regarded as a safe in-office procedure. Although systemic reactions can occur, they are uncommon, and severe or life-threatening outcomes are exceedingly rare when recommended techniques and safety precautions are followed. The most convincing risk factor reported is the use of PtP testing with fresh multiple cross-reactive food allergens. Therefore, clear indications for choosing PtP versus SPT with commercial extracts, along with performing PtP in multiple sessions for at-risk individuals, are highly recommended. Moreover, clinicians should be prepared for the prompt recognition and treatment of systemic reactions.

## Data Availability

The datasets presented in this article are not readily available because no dataset. Requests to access the datasets should be directed to carla.mastrorilli@gmail.com.
